# Intratumoral Morphological Heterogeneity of Breast Cancer As an Indicator of the Metastatic Potential and Tumor Chemosensitivity

**Published:** 2017

**Authors:** T.S. Gerashchenko, M.V. Zavyalova, E.V. Denisov, N.V. Krakhmal, D.N. Pautova, N.V. Litviakov, S.V. Vtorushin, N.V. Cherdyntseva, V.M. Perelmuter

**Affiliations:** Cancer Research Institute, Tomsk National Research Medical Center, Russian Academy of Sciences, Kooperativny per., 634050, Tomsk, Russia; Tomsk State University, 36 Lenin Ave., Tomsk, 634050, Russia; Siberian State Medical University, Moskovsky trakt 2, Tomsk, 634050, Russia

**Keywords:** intratumoral heterogeneity, invasion, metastasis, breast cancer, chemotherapy, epithelial-mesenchymal transition

## Abstract

Breast cancer (BC) demonstrates considerable intratumoral morphological heterogeneity. The aim of this work was to evaluate the relationship among different morphological structures, the rate of metastasis, and efficacy of neoadjuvant chemotherapy (NAC) in NAC-treated (*n *= 427) and NAC-naïve (*n *= 249) BC patients. We also studied the involvement of an epithelial-mesenchymal transition (EMT) in the development of the intratumoral morphological heterogeneity of BC. We found a significant association between the intratumoral morphological heterogeneity and the rate of BC metastasis and response to NAC, which, in most cases, correlated with the presence of alveolar and trabecular structures. In particular, the rate of lymph node metastasis in tumors containing alveolar and trabecular structures was higher compared to that in tumors lacking such structures. NAC-treated patients with alveolar and trabecular structures had a high distant metastasis rate and a low metastasis-free survival rate. Furthermore, alveolar and trabecular structures were found to be associated with a lack of response to NAC. Interestingly, the association between alveolar structures and a high distant metastasis rate was found only in NAC-unresponsive patients, whereas the association between trabecular structures and an increased distant metastasis was revealed in responders. Alveolar structures were associated with chemoresistance only in patients with lymph node metastases, whereas trabecular structures were associated with chemoresistance only in patients without lymph node metastases. In general, increased intratumoral morphological diversity correlated with considerable chemoresistance and a high metastasis rate of BC. We found variable expressions of epithelial (*EPCAM *and *CDH1*) and mesenchymal (*ITGA5*, *ITGB5*, *CDH2*, *CDH11*, *TGFb2*, *ZEB1*, *MMP2*, *DCN*, *MST1R*) markers and, thus, different EMT manifestations in different morphological structures. Therefore, intratumoral morphological heterogeneity of BC may serve as an indicator of the metastatic potential and tumor chemosensitivity.

## INTRODUCTION

One of the main characteristics of malignant tumors is the heterogeneity of their cell composition, or intratumoral heterogeneity. The heterogeneity of the cell shape and the morphology within the tumor was first described by Rudolf Virchow in the 19th century [1]. Since the time of Virchow’s work, the concept of intratumoral heterogeneity has been greatly advanced. Different cell populations are now known to be able to coexist in the tumor and specifically affect the tumor’s biological behavior [2]. A high degree of intratumoral heterogeneity is associated with a poor clinical prognosis, and the presence of certain cell populations is associated with metastasis and the development of tumor drug resistance [3]. The investigation of various types of intratumoral heterogeneity and the features of its impact on the clinical course of malignancies is one of the major challenges of modern oncology.

Breast tumors are characterized by a significant variability of the cell composition, as well as by histologic, expression, and genotypic heterogeneity [4]. The intratumoral morphological heterogeneity has been described in invasive breast carcinoma of no special type that is the most common histological type of BC (occurrence rate of up to 80%). According to the WHO classification, breast cancer cells can be arranged in cords, clusters, and trabeculae; in some tumors, a solid or syncytial cell component is prevalent [5]. Furthermore, breast tumors can include tubular, alveolar, glandular-papillary, and solid structures of tumor cells, as well as carcinoid-like areas or scirrhous foci [6]. In our previous studies, we primarily focused on the investigation of tubular, alveolar, solid, and trabecular structures, as well as discrete groups of tumor cells, their genetic and expression portrait, and their association with the rate of lymph node metastasis and the efficacy of neoadjuvant chemotherapy of BC [7-12].

Previously, we had assumed that different structures might be a morphological manifestation of an invasive growth of breast tumors [8, 13]. For example, individual and collective cell invasion patterns can be conventionally distinguished. They are represented by various patterns differing from each other in their form and mechanisms of cell migration. Individual migration can occur both as an amoeboid motion via actomyosin-driven contractions of the cytolemma and as a mesenchymal (fibroblast-like) motion associated with an elongation of the cell’s shape, enhanced adhesion of cells to the extracellular matrix, and an increased proteolytic activity. Collective invasion occurs via a mesenchymal migration of tumor cell groups (clusters, solid structures, etc.) [13, 14]. The key mechanism of cell migration is a EMT-related transformation of epithelial cells into mesenchymal cells and the acquisition of a locomotor phenotype by the latter [15]. During EMT, epithelial cells lose cell-cell interactions and the apical-basal polarity and acquire an elongated shape and mobility, which enables them to detach from the primary tumor. These changes are regulated by the Snail, Twist, Slug, ZEB1, and ZEB2 transcription factors and are accompanied by a loss of cell-cell adhesion molecules (E-cadherin, EpCAM, etc.) and by the acquisition of mesenchymal features, such as the expression of N-cadherin, vimentin, etc. [14, 16-19]. Invasive growth is known to be closely related to the metastasis process and directly affect the development of resistance to drugs [15].

In this paper, we consider the relationship between the rate of lymph node and distant metastasis, as well as the efficacy of treatment and the different morphological structures present in a primary breast tumor. We analyze the expression patterns of EMT-associated genes in different morphological structures to understand the involvement of the invasion process in the development of the intratumoral morphological heterogeneity of BC.

## MATERIALS AND METHODS

**Morphological analysis and association studies**

We analyzed two groups of BC patients treated at the Department of General Oncology of the Cancer Research Institute, Tomsk National Research Medical Center. The first group consisted of 427 patients with invasive breast carcinoma of no special type (T_1–4_N_0–3_M_0–1_), aged 28 to 90 years (mean age, 49.9 ± 9.44 years), who received 2–4 NAC courses using CMX/CMF, CAX, FAC, and taxane regimens. The second group included 249 patients with invasive breast carcinoma of no special type (T_1–4_N_0–3_M_0–1_), aged 21 to 85 (mean age, 56.02 ± 11.16 years), who did not receive NAC. The characteristics of the patients are presented in *Table 1* and *Table 2*.

**Table 1:** Clinicopathological characteristics of NAC-treated patients

Clinicopathological parameter	Indicator	Number of cases, %
Age	≤ 50 years	230 (53.8)
	> 50 years	197 (46.2)
Menopause	Premenopausal	224 (52.4)
	Postmenopausal	203 (47.6)
Tumor size	T_1_	101 (23.7)
	T_2_	266 (62.3)
	T_3_	48 (11.2)
	T_4_	12 (2.8)
Lymph node metastases	N_0_	213 (49.9)
	N_1_	138 (32.3)
	N_2_	64 (15.0)
	N_3_	12 (2.8)
Distant metastases	M_0_	220 (51.5)
	M_1_	127 (29.7)
	No data	80 (18.8)
Expression of estrogen receptors	Yes	167 (39.1)
	No	158 (37.0)
	No data	102 (23.8)
Expression of progesterone receptors	Yes	154 (36.1)
	No	173 (40.5)
	No data	100 (23.4)
Expression of epidermal growth factor receptors (HER2)	Yes	48 (19.3)
	No	201 (80.7)
Expression of epidermal growth factor receptors (HER2)	-	160 (37.5)
	+	76 (17.9)
	++	26 (6.0)
	+++	8 (1.8)
	No data	157 (36.8)
Molecular subtype	Luminal	153 (35.8)
	Triple-negative	96 (22.5)
	HER2-positive	26 (6.1)
	No data	152 (35.6)
NAC regimen	CMX/CMP	165 (38.7)
	CAX	56 (13.1)
	Taxanes	31 (7.2)
	FAC	110 (25.8)
	No data	65 (15.2)
NAC efficacy	Complete response	27 (6.3)
	Partial response	183 (42.9)
	Stable disease	133 (31.2)
	Progressive disease	21 (4.9)
	No data	63 (14.7)

Notes: CMX – cyclophosphamide, methotrexate, and Xeloda; CMF – cyclophosphamide, methotrexate, and 5-fluorouracil; FAC – 5-fluorouracil, adriblastina, and cyclophosphamide; CAX – cyclophosphamide, adriblastina, and Xeloda; taxanes – docetaxel and paclitaxel.

Morphological structures were investigated in biopsy and surgical specimens from patients of both groups. Biopsy and surgical material, which was provided in two to six specimens of a breast tumor, was used to prepare two sections (5–6 μm). The sections were stained with hematoxylin and eosin and examined by two or three pathologists for the presence of different morphological structures (tubular, alveolar, solid, and trabecular structures, as well as discrete groups of tumor cells) in accordance with [10].

**Table 2:** Clinicopathological characteristics of NAC-naive patients

Parameter	Indicator	Number of cases, %
Age	≤ 50 years	77 (31.0)
	> 50 years	172 (69.0)
Menopause	Premenopausal	169 (67.9)
	Postmenopausal	80 (32.1)
Tumor size	T_1_	145 (58.2)
	T_2_	95 (38.2)
	T_3_	8 (3.2)
	T_4_	1 (0.4)
Lymph node metastases	N_0_	146 (58.6)
	N_1_	65 (26.1)
	N_2_	26 (10.5)
	N_3_	12 (4.8)
Distant metastases	M_0_	222 (89.2)
	M_1_	27 (10.8)
Expression of estrogen receptors	Yes	184 (74.0)
	No	65 (26.0)
Expression of progesterone receptors	Yes	159 (63.9)
	No	90 (36.1)
Molecular subtype	Luminal	195 (78.3)
	Triple-negative	36 (14.5)
	HER2-positive	17 (6.8)
	No data	1 (0.4)

The NAC efficacy was assessed based on the results of instrumental studies (ultrasound and mammography) using the RECIST scale [20]. Patients were classified into responders that show a response (complete or partial) to NAC and non-responders with a lack of response (a stable or progressive disease).

We performed association studies of the relationship between different morphological structures of tumors and the clinical parameters of the disease in both groups of patients, as well as the efficacy of chemotherapy in the NAC group. The obtained data were processed with the χ2 test and Fisher’s exact test using Statistica 8.0. Survival was evaluated using the Kaplan-Meier method. The results were considered significant at *p * < 0.05.

**Expression analysis**

**Fig. 1:**
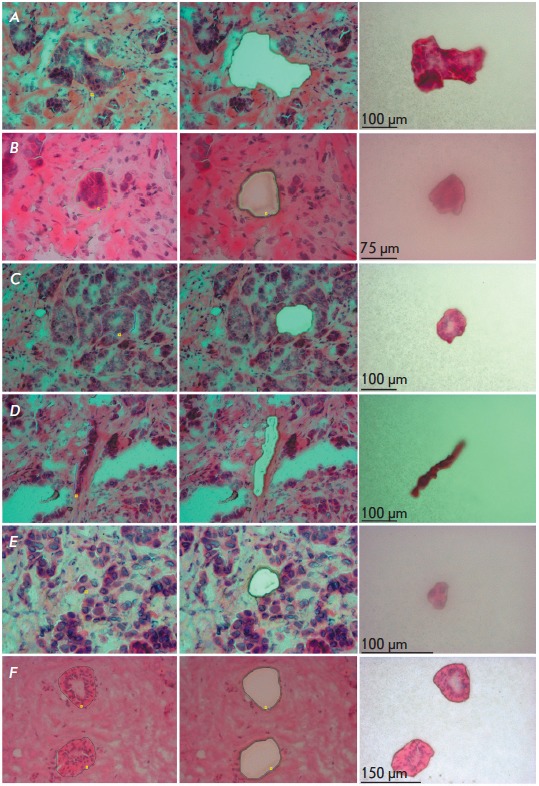
Laser microdissection of different morphological structures and morphologically intact ducts from tumor and normal tissues of the breast, respectively. The figure shows sections before and after microdissection, as well as the microdissected structures on adhesive caps: *A *– solid structure, *B *– alveolar structure, *C *– tubular structure, *D *– trabecular structure, *E *– a discrete group of tumor cells, *F *– morphologically intact ducts. Hematoxylin and eosin staining

Expression of the genes involved in EMT (*EPCAM*, *ITGA5*, *ITGB5*, *CDH1*, *CDH2*, *CDH11*, *TGFb2*, *ZEB1*, *MMP2*, *DCN*, and *MST1R*) was analyzed in different morphological structures of the breast tumors by quantitative real-time PCR. We used frozen samples of tumor tissue obtained during surgery from seven patients with invasive breast carcinoma of no special type, luminal B molecular subtype (T_1–2_N_0–3_M_0_), aged from 42 to 65 years (mean age, 56.42 ± 8.75 years), who did not receive NAC. Hematoxylin- and eosin-stained sections of freshly frozen breast tumor specimens were used to isolate five types of morphological structures (*Fig. 1*) using a PALM laser microdissection (Carl Zeiss, Germany) according to the previously published procedure [8, 21]. In particular, we obtained tubular, alveolar, and trabecular structures (90–120 samples, ~ 900–1,200 cells), solid structures (50– 60 samples, up to 5,000 cells), and discrete groups of tumor cells (300–350 samples, ~ 400–600 cells). To prevent the occurrence of stromal components in the samples, laser microdissection was performed along the edge of the outer epithelial layer of the morphological structures. The microdissected samples were used to isolate total RNA (RNeasy Micro Plus Kit, Qiagen, USA). The RNA integrity (RIN) was assessed using the 2200 TapeStation instrument (Agilent, USA). The RNA was subjected to reverse transcription (cDNA), ligation, and whole transcriptome amplification (QuantiTect Whole Transcriptome Kit, Qiagen, USA). The amplified cDNA was used for PCR according to [8]. The expression analysis results were evaluated relative to morphologically intact breast ducts that were also sampled during the laser microdissection of normal tissue adjacent to the tumor and the reference gene *ACTB *using the Pfaffl method [22].

## RESULTS

Chemotherapy is well known to affect the structure of the tumor population. Chemotherapy-induced changes in tumor cells largely determine the further course of the disease: drug resistance, metastasis, and recurrence [23-25]. In this regard, we analyzed two independent samples of BC patients with and without NAC treatment.

**The rate of lymph node and distant metastasis in NAC-naïve BC patients, depending on intratumoral morphological heterogeneity**

**Fig. 2:**
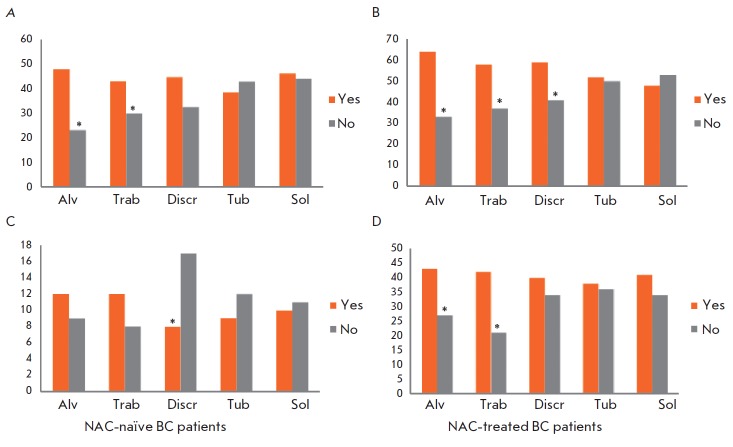
The rate of lymph node (*A*, *B*) and distant (*C*, *D*) metastasis in BC patients, depending on the presence of different morphological structures in tumors. *A *– the rate (%) of lymph node metastasis in NAC-naïve patients. *B *– the rate (%) of lymph node metastasis in NAC-treated patients. *C *– the rate (%) of distant metastasis in NAC-naïve patients. *D *– the rate (%) of distant metastases in NAC-treated patients. Alv – alveolar structures; Trab – trabecular structures; Discr – discrete groups of tumor cells; Tub – tubular structures; Sol – solid structures. * – Statistically significant differences (*p* < 0.05)

The presence of either alveolar or trabecular structures in the tumors of NAC-naive patients was associated with a higher rate of lymph node metastasis of BC compared to tumors lacking those structures (47.8 vs. 23.2%, *p *= 0.0004; 43.0 vs. 30.0%, *p *= 0.0012, respectively; *Fig. 2A*). The absence of discrete groups of cells in the tumors was associated with an increased rate of distant metastasis compared to the tumors containing the structures (16.9 vs. 8.2%, *p *= 0.043; *Fig. 2B*). These findings are in general consistent with the data, which were previously obtained in a smaller sample of patients, on the association between alveolar structures and the rate of lymph node metastasis [7, 10, 11], as well as the association between trabecular structures and the risk of lymph node metastasis [12].

**The rate of lymph node metastasis in NAC-treated BC patients, depending on intratumoral morphological heterogeneity**

**Fig. 3:**
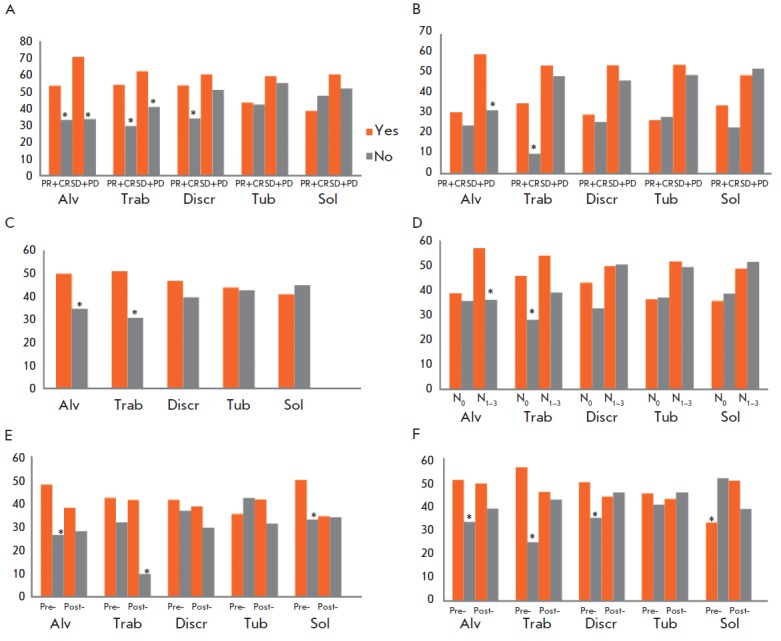
The chemotherapy efficacy and metastasis rate in NAC-treated BC patients, depending on the presence of different morphological structures in tumors. *A, B *– the rate (%) of lymph node and distant metastasis in responders and nonresponders to neoadjuvant chemotherapy. *C *– the frequency (%) of a lack of response to neoadjuvant chemotherapy. *D *– the frequency (%) of a lack of response to neoadjuvant chemotherapy in patients with/without lymph node metastases. *E, F *– the rate (%) of distant metastasis and the frequency (%) of a lack of response to neoadjuvant chemotherapy in pre- and postmenopausal patients. Alv – alveolar structures; Trab – trabecular structures; Discr – discrete groups of tumor cells; Tub – tubular structures; Sol – solid structures; Pre- – premenopausal patients; Post- – postmenopausal patients; N0 – negative lymph node status; N1_–3_ – positive lymph node status; PR+CR – partial response and complete response; SD+PD – stable disease and progressive disease. * – Statistically significant differences (*p* < 0.05)

In NAC-treated patients, the rate of lymph node metastasis of tumors containing alveolar or trabecular structures or discrete groups of cells was higher than that in tumors lacking these structures (64.2 vs. 33.0%, *p* < 0.0001; 57.7 vs. 36.8%, *p* < 0.0001; 59.3 vs. 41.4%, *p *= 0.0002, respectively; *Fig. 2B*). We previously de scribed a relationship between alveolar structures in the tumors of NAC-treated patients and an increased risk of lymph node metastasis. However, the study group was small, and that relationship was observed only in postmenopausal patients [26]. In patients with alveolar or trabecular structures, the lymph node metastasis rate was higher, regardless of the efficacy of chemotherapy (*p *= 0.0032 and *p* < 0.0001; *p *= 0.0004 and *p *= 0.0152; *Fig. 3A*). On the contrary, in the group of responders, the rate of lymph node metastasis was higher in patients with discrete groups of cells in the tumor than in patients with tumors lacking such structures (53.8 vs. 34.0%, *p *= 0.0041, respectively; *Fig. 3A*).

**The rate of distant metastasis in NAC-treated BC patients, depending on intratumoral morphological heterogeneity**

**Fig. 4:**
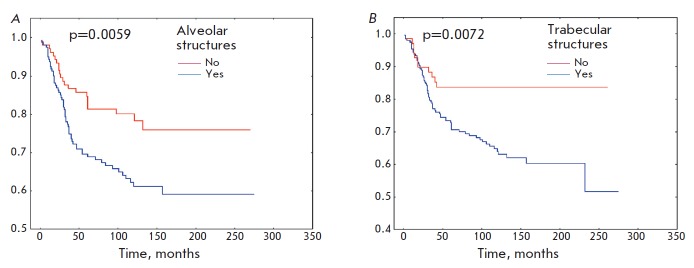
Metastasis-free survival rate of BC patients with alveolar (*A*) and trabecular (*B*) structures in tumors

In patients with alveolar or trabecular structures in tumors, the rate of distant metastasis was higher than that in patients with tumors lacking such structures (42.8 vs. 27.3%, *p *= 0.0036; 41.9 vs. 20.7%, *p *= 0.0005; *Fig. 2D*). Furthermore, patients with alveolar or trabecular structures had a low metastasis-free survival rate compared to patients lacking these structures (*p *= 0.0087 and *p *= 0.0073, respectively; *Fig. 4 A,B*). A relationship between alveolar structures and distant metastasis was found only in non-responders to chemotherapy (58.5 vs. 31.0%, *p *= 0.0030; *Fig. 3B*), whereas in the case of trabecular structures, this relationship was observed only in responders (34.3 vs. 9.3%, *p *= 0.0011; *Fig. 3B*). In addition, the relationship between morphological structures and distant metastasis depended on the menopausal status. For example, alveolar and solid structures were associated with a high rate of distant metastasis only in premenopausal patients (48.0 vs. 26.5%, *p *= 0.0059; 50.0 vs. 33.0%, *p *= 0.028, respectively; *Fig. 3E*), while trabecular structures were associated with a high risk of distant metastasis only in a subgroup of postmenopausal patients (41.4 vs. 9.7%, *p *= 0.0002; *Fig. 3E*).

**The dependence of the NAC efficacy on the intratumoral morphological heterogeneity of BC**

The presence of alveolar or trabecular structures in breast tumors was more frequently associated with a lack of response to NAC (a stable or progressive disease) compared to tumors lacking these structures (50.3 vs. 35.8%, *p *= 0.0056; 50.7 vs. 31.6%, *p *= 0.0004, respectively; *Fig. 3B*). Earlier, in a smaller sample of patients, we described a relationship between alveolar and trabecular structures and the chemoresistance of BC and examined the possible mechanisms behind this relationship [8, 9]. Interestingly, a relationship between alveolar structures and chemoresistance was observed only in a group with lymph node metastases: 57.1 (patients with structures) vs. 36.2% (patients without structures; *p *= 0.0089, respectively; *Fig. 3D*). At the same time, a relationship between trabecular structures and chemoresistance was found only in patients without lymph node metastases (45.8 vs. 28.0%, *p *= 0.0117, respectively; *Fig. 3D*). The relationship between structures and the NAC efficacy also depended on the menopausal status of BC patients. The presence of alveolar or trabecular structures or discrete groups of cells was associated with a lack of response to chemotherapy only in a subgroup of premenopausal patients (51.0 vs. 33.3%, *p *= 0.0133; 56.2 vs. 24.7%, *p *= 0.0000; 50.0 vs. 35.0%, *p *= 0.0365, respectively; *Fig. 3F*). The presence of solid structures was associated with a response to chemotherapy only in premenopausal patients (67.0 vs. 48.4%, *p *= 0.0094; *Fig. 3F*).

**Fig. 5:**
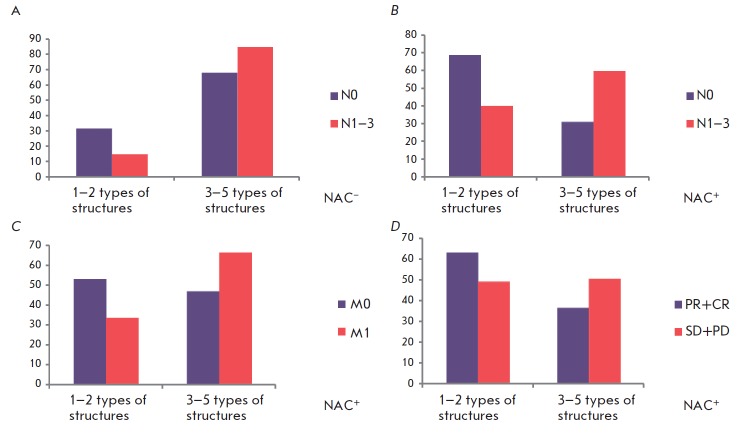
The chemotherapy efficacy and metastasis rate depending on the number of different morphological structures in breast tumors. *A *– the rate (%) of lymph node metastasis in NAC-naive patients with low (1–2 types of structures) and high (3–5 types of structures) intratumoral morphological diversity. *B, C *– the rate (%) of lymph node and distant metastasis in NAC-treated patients with low (1–2 types of structures) and high (3–5 types of structures) intratumoral morphological diversity. *D *– the frequency (%) of a lack of response to NAC in patients with low (1–2 types of structures) and high (3–5 types of structures) intratumoral morphological diversity. NAC^–^ – NAC-naive patients; NAC^+^ – NAC-treated patients. Only statistically significant differences (*p* < 0.05) are shown

It should be noted that drug resistance and a high rate of lymph node and distant metastasis were in general more often observed in tumors with three to five types of morphological structures than in tumors with one or two types (*p *= 0.0082; *p* < 0.0001; *p* = 0.0005, respectively; *Fig. 5*).

An analysis of the association between intratumoral morphological heterogeneity and the metastasis rate and NAC efficacy, depending on chemotherapy regimens, molecular subtypes of BC, and tumor size, was not performed due to a disproportionate ratio of case numbers in subgroups.

**Expression of EMT genes in different morphological structures of breast tumors**

**Fig. 6:**
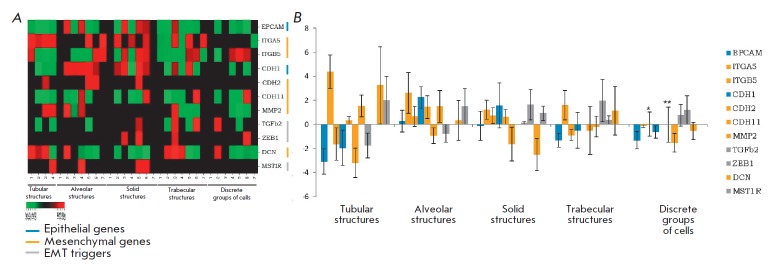
Expression of EMT genes in different morphological structures of breast tumors. *A *– a heat map of the expression level. *B *– the mean log expression level. ^*^ – an expression level of 0.029. ^**^ – an expression level of 0.016. The large standard error of the mean is due to the small sample size, significant variation in expression levels among patients, and the absence of gene expression in some samples of the morphological structure

An expression analysis of the genes involved in EMT demonstrated that each of the structures was associated with a specific set of markers (*Fig. 6*). Expression of epithelial genes, *EPCAM* and *CDH1*, was typical of all structures (*Fig. 6B*). However, tubular and trabecular structures and discrete groups of tumor cells had low expression levels (*Fig. 6B*). Expression of EMT triggers, *TGFb2*, *ZEB1*, and *MST1R*, was heterogeneous both in different breast tumors and in the structures (*Fig. 6A*). *ZEB1* and *MST1R *were more often expressed in solid structures, while *TGFb2 *was more often expressed in trabecular structures. Mesenchymal genes (*CDH2*, *CDH11*, *ITGA5*, *ITGB5*, *MMP2*, and *DCN*) had variable expression levels. *CDH2 *encoding a classical mesenchymal marker, N-cadherin, was not expressed in most of the structures. Expression of the integrin alpha 5 gene (*ITGA5*) was almost completely absent in discrete groups of tumor cells and was more pronounced in tubular and trabecular structures. On the contrary, the *ITGB5 *gene, along with the decorin proteoglycan gene (*DCN*), was uniformly expressed in different structures. Interestingly, the matrix metalloproteinase 2 gene (*MMP2*) was not expressed solely in solid structures (*Fig. 6A*).

## DISCUSSION

Our findings demonstrate the differential contribution of different morphological structures to chemoresistance and the progression of BC. A lymph node and distant dissemination of tumor cells is mainly associated with alveolar and trabecular structures. Interestingly, a contribution of alveolar and trabecular structures to distant metastasis, as well as an impact of discrete groups of cells on the rate of lymph node metastasis, was observed only in chemotherapy-treated patients. Obviously, chemotherapy is a factor that modulates BC progression. Noteworthy, the presence of alveolar and trabecular structures in the tumors of NAC-treated patients was simultaneously associated with both the rate of lymph node and distant metastasis and chemoresistance, which suggests a relationship between these processes. The relationship between alveolar and trabecular structures and the rate of lymph node metastasis did not depend on the efficacy of chemotherapy. An association of alveolar structures with the rate of distant metastasis was observed only in non-responders, and an association of trabecular structures with the rate of distant metastasis was present in patients with a response to NAC. The dependence of a high rate of lymph node and distant metastasis and drug resistance on a greater morphological diversity of the tumor may also be explained by an increased proportion of alveolar and trabecular structures in the total amount of morphological components. Intratumoral morphological heterogeneity was demonstrated to significantly affect the clinical outcome of BC. This was evidenced in the observation that the survival rate of patients with alveolar or trabecular structures in tumors was significantly lower than that of patients without these structures.

We had previously suggested that different morphological structures could be correlated with invasive growth patterns: discrete groups of cells are associated with individual migration, while solid, alveolar, trabecular, and tubular structures are associated with different types of collective migration [8, 13]. During invasive growth, tumor cells migrate by means of intravasation from the primary site to the lymph and/or blood vessels, followed by dissemination to other organs. Neoplastic cells lack a programmed ability to migrate; they acquire the ability via a triggering of signaling cascades *de novo *or a response to cytokine stimulation or under the influence of antitumor therapy. EMT, as a central molecular program induced during mesenchymal cell migration, creates the preconditions for the development of at least three phenotypic states of tumor cells: the epithelial, mesenchymal, and intermediate epithelial-mesenchymal phenotypes [27, 28]. The most aggressive intermediate state is that where the cell has a hybrid phenotype, acquiring mesenchymal properties and partially preserving epithelial features [27-29]. Furthermore, a hybrid EMT state was shown *in vitro *to be associated with an increased formation of spheroids or tumor cell clusters (2–50 cells) capable of intravasation into blood vessels and associated with more severe metastasis [27, 28]. Interestingly, the shape and amount of cells in small alveolar structures are similar to those in the spheroids circulating in the blood of cancer patients, and according to our findings, the presence of alveolar structures in tumors is associated with an increased rate of lymph node and distant metastasis. Alveolar structures, along with trabecular ones, are likely to be collective migration types with a hybrid epithelial-mesenchymal phenotype, which provides the structures with aggressive features and, as a consequence, high metastatic activity. Indeed, based on an evaluation of the expression of epithelial and mesenchymal state genes, alveolar and trabecular structures were found to be characterized by an intermediate EMT state, with the epithelial (*EPCAM*, *CDH1*) and mesenchymal features (*CDH11*, *ITGB5*, *MMP2*, *DCN*, etc.) preserved. Solid structures may also be considered as an intermediate state of EMT, but with a predominance of the epithelial features (*EPCAM*, *CDH1*). For example, solid structures may represent a type of collective migration with partial EMT in the invasive front area [13, 14]. The relationship between alveolar structures and increased distant metastasis may also be explained by an involvement of these morphological structures in the formation of premetastatic niches through a high production of cytokines and growth factors [30].

Interestingly, tubular structures were characterized by a low expression of epithelial genes, *EPCAM *and *CDH1*, along with an increased expression of mesenchymal genes, *DCN*, *ITGA5*, and *MMP2*. The morphology of tubular structures, which resembles that of normal breast ducts, rather points to the epithelial nature of these morphological structures. Furthermore, the presence of tubular structures is routinely used as a favorable prognostic indicator in the assessment of a tumor grade: the larger the number of tubular structures in the tumor, the lower grade it is, and vice versa [31]. Our previous findings likewise confirm the positive predictive value of tubular structures: an increased ratio of trabecular and tubular structures (Tr/Tub) in tumors is associated with a high rate of distant metastasis. For example, the risk of distant metastasis for a Tr/ Tub ratio of 2 is 5-fold higher than that for Tr/Tub of 1 [32]. Probably, the expression of mesenchymal markers in tubular structures may be explained by the fact that part of the structures undergoes initial EMT. Furthermore, there is evidence suggesting that epithelial cells within a heterogeneous tumor may maintain the transition state of other tumor cells undergoing EMT through the secretion of stimulating factors [19].

Discrete groups of tumor cells as individual migration patterns are the most compositionally heterogeneous morphological components of a tumor; they may contain single cells or groups of cells likely capable of both mesenchymal and amoeboid motion. The phenotype of discrete groups of tumor cells has a variable expression portrait with a low representation of epithelial markers on the background of slightly increased mesenchymal features. This is somewhat surprising, because single tumor cells or their small clusters arise likely due to EMT when cells lose their epithelial features and the ability to form multicellular clusters. At the same time, it is emphasized that the use of known markers of the mesenchymal phenotype, some of which we have used in our work, is not sufficient to judge about the presence or absence of mesenchymal features in tumor cells [33].

There is ample evidence that preoperative chemotherapy is able to modify the genome of tumor cells and affect the tumor population structure [23-25]. The molecular profile of primary tumor samples of triple-negative BC after NAC was shown to differ from the profile of biopsy samples of the same tumors before chemotherapy [34]. Chemotherapy was found to be able to initiate the development and/or expansion of cell populations resistant to treatment [24]. Under the influence of NAC, the morphological structures of breast tumors may acquire additional features that enhance tumor dissemination and promote a subsequent development of chemoresistance. Furthermore, drug resistance and invasion are closely interrelated processes that support each other during malignant growth [15, 35, 36]. This relationship is obvious in the case of the alveolar and trabecular structures associated simultaneously with both a high rate of lymph node and distant metastasis of the tumor and resistance to therapy. The point is that the signaling pathways common to invasion and chemoresistance are activated in migrating cells. The cascade triggered by integrins, cadherins, and small GTPases Rac and Rho intersects with PI3K, mTOR, Src, and MAP-kinase pathways [15, 35]. The EMT state of migrating cells reduces the sensitivity of the cells to antitumor therapy [18, 24]. Tumor cells undergoing EMT exhibit high resistance to radiotherapy and some chemotherapeutic agents [24]. In addition, EMT induces ABC-transporters and activates the alternative oncogenic signaling pathways EGFR, RAF, and MEK, which promotes the development of resistance to therapy, in particular to targeted therapy [37, 38].

Therefore, the biological behavior of a tumor largely depends on the features of its invasive growth [14, 15]. We have demonstrated that the intratumoral morphological heterogeneity of BC, which is probably represented by invasive growth patterns at various EMT stages, may be a factor that determines the metastatic tumor potential and the ability of cells to respond to treatment and affect the clinical outcome of the disease.

## CONCLUSION

The main obstacle to a successful diagnosis and treatment of cancers is the intratumoral heterogeneity. Because of significant intratumoral diversity, modern biopsy-based diagnostic techniques do not provide a full understanding of the further clinical manifestations of the tumor. We have demonstrated that the intratumoral morphological heterogeneity of BC probably represented by invasive tumor growth patterns is associated with the rate of lymph node and distant metastasis and the efficacy of preoperative therapy. Probably, the morphological diversity of a tumor may form the basis for the creation of an effective model for developing prognostic and predictive criteria for breast cancer, while alveolar and trabecular structures, as the key indicators of aggressive tumor growth, may become targets in the development of targeted therapy.

## Abbreviations

NAC: neoadjuvant chemotherapy
BC: breast cancer
EMT: epithelial-mesenchymal transition
